# Identification of a unique hepatocellular carcinoma line, Li-7, with CD13(+) cancer stem cells hierarchy and population change upon its differentiation during culture and effects of sorafenib

**DOI:** 10.1186/s12885-015-1297-7

**Published:** 2015-04-11

**Authors:** Takeshi Yamada, Masato Abei, Inaho Danjoh, Ryoko Shirota, Taro Yamashita, Ichinosuke Hyodo, Yukio Nakamura

**Affiliations:** 1 Division of Gastroenterology, Faculty of Medicine, University of Tsukuba, 1-1-1 Tennoudai, Tsukuba, Ibaraki 305-8575 Japan; 2 Cell Engineering Division, RIKEN BioResource Center, 3-1-1 Koyadai, Tsukuba, Ibaraki 305-0074 Japan; 3 Functional Evaluation of Genomic Polymorphisms, Tohoku Medical Megabank Organization, Sendai, Japan; 4 Department of Gastroenterology, Kanazawa University Hospital, 13-1, Takara-machi, Kanazawa, Ishikawa 920-8641 Japan

**Keywords:** Cancer stem cell, Hepatocellular carcinoma, CD13, CD166, Sorafenib, Population change

## Abstract

**Backgrounds:**

Cancer stem cell (CSC) research has highlighted the necessity of developing drugs targeting CSCs. We investigated a hepatocellular carcinoma (HCC) cell line that not only has CSC hierarchy but also shows phenotypic changes (population changes) upon differentiation of CSC during culture and can be used for screening drugs targeting CSC.

**Methods:**

Based on a hypothesis that the CSC proportion should decrease upon its differentiation into progenitors (population change), we tested HCC cell lines (HuH-7, Li-7, PLC/PRF/5, HLF, HLE) before and after 2 months culture for several markers (CD13, EpCAM, CD133, CD44, CD90, CD24, CD166). Tumorigenicity was tested using nude mice. To evaluate the CSC hierarchy, we investigated reconstructivity, proliferation, ALDH activity, spheroid formation, chemosensitivity and microarray analysis of the cell populations sorted by FACS.

**Results:**

Only Li-7 cells showed a population change during culture: the proportion of CD13 positive cells decreased, while that of CD166 positive cells increased. The high tumorigenicity of the Li-7 was lost after the population change. CD13(+)/CD166(−) cells showed slow growth and reconstructed the bulk Li-7 populations composed of CD13(+)/CD166(−), CD13(−)/CD166(−) and CD13(−)/CD166(+) fractions, whereas CD13(−)/CD166(+) cells showed rapid growth but could not reproduce any other population. CD13(+)/CD166(−) cells showed high ALDH activity, spheroid forming ability and resistance to 5-fluorouracil. Microarray analysis demonstrated higher expression of stemness-related genes in CD166(−) than CD166(+) fraction. These results indicated a hierarchy in Li-7 cells, in which CD13(+)/CD166(−) and CD13(−)/CD166(+) cells serve as slow growing CSCs and rapid growing progenitors, respectively. Sorafenib selectively targeted the CD166(−) fraction, including CD13(+) CSCs, which exhibited higher mRNA expression for *FGF3* and *FGF4*, candidate biomarkers for sorafenib. 5-fluorouracil followed by sorafenib inhibited the growth of bulk Li-7 cells more effectively than the reverse sequence or either alone.

**Conclusions:**

We identified a unique HCC line, Li-7, which not only shows heterogeneity for a CD13(+) CSC hierarchy, but also undergoes a “population change” upon CSC differentiation. Sorafenib targeted the CSC *in vitro*, supporting the use of this model for screening drugs targeting the CSC. This type of “heterogeneous, unstable” cell line may prove more useful in the CSC era than conventional “homogeneous, stable” cell lines.

**Electronic supplementary material:**

The online version of this article (doi:10.1186/s12885-015-1297-7) contains supplementary material, which is available to authorized users.

## Background

For a long time, tumor progression was explained on the basis of a stochastic model in which every cancer cell in a tumor could repopulate the entire tumor mass. However, a paradigm shift occurred recently and a new hierarchical model achieved wide acceptance: under this model, a minority of the tumor cells acts as cancer stem cells (CSCs) or tumor-initiating cells to give rise to the entire tumor mass. CSCs are supposed to possess the capacity for self-renewal and the hierarchical generation of heterogeneous cancer cells within tumor tissues [1]. Slow-growing CSCs, which are at the top of this hierarchy, are resistant to conventional chemotherapy or radiotherapy and account for the progression, metastasis and recurrence of cancers [2,3]. This new CSC model has deepened our understanding of the complexity of tumor tissues [4].

Hepatocellular carcinoma (HCC) is one of the major causes of cancer-related mortality worldwide, with especially high prevalence in East Asian countries [5]. A range of therapeutic options is currently available for HCC depending on the clinical stage of the disease [6]. However, the only available drug for advanced stage HCC is sorafenib, an orally active multi-kinase inhibitor that targets serine and threonine kinases (B-RAF), and tyrosine kinases (VEGFR, PDGFR, FLT-3, c-KIT); however, the drug has limited efficacy [7,8]. Currently, there is considerable interest in developing more effective therapeutic strategies, especially for advanced stage HCC patients. In studies of HCC, CSCs were identified as a side population fraction [9,10], or as cells expressing CD133 [11,12], CD90 [13], EpCAM [14], CD44 [15], or CD24 [16], or by an aldefluor assay [17]. More recently, CD13 was reported to be a marker for CSCs that were semi-quiescent, more immature stem-like, or dormant [18]. In addition, CSCs for HCC have been visualized by their low levels of proteasome and reactive oxygen species (ROS) [19].

One of the lasting problems associated with the previous paradigm was that cancer cell lines were regarded as ideal for research if they were “homogeneous and stable” as long as they are free from misidentification and cross contamination [20]. Consequently, many cell lines deposited in cell banks had been cultured and passaged for more than 6 months in order to ensure the cells showed these characteristics. Thus, these cell lines are likely to be less than ideal for cancer research under the current CSC paradigm and might produce results that are very different from clinical samples. Recent studies on a number of cancer cell lines have identified the expected “heterogeneity”; however, since many of these cell lines are “stable”, the differentiation of CSCs cannot easily be evaluated *in vitro*. Additionally, although it is well recognized that new therapeutic strategies need to be developed, the screening of drugs that target CSCs is hampered by the limited number of *in vitro* models that display a clear CSC hierarchy, and allow discrimination of slow-growing CSCs from their rapidly-growing progenitors.

We hypothesized that an unstable cell line that changes its phenotype upon differentiation of CSCs during culture (a population change) might provide an improved *in vitro* model for HCC. Based on this hypothesis, we screened HCC cell lines to identify those that not only maintain a clear CSC hierarchy but also undergo population changes; we then investigated the value of such cell lines for screening drugs targeting CSC. We assumed that if a cell line contained a slow-growing CSC subpopulation, the relative size of this subpopulation would decrease during culture because of its slow growth and its differentiation into rapid-growing progenitors (population change). In the present study, we tested several HCC cell lines (HuH-7, Li-7, PLC/PRF/5, HLF, HLE) using a range of markers (CD13, EpCAM, CD133, CD44, CD90, CD24, CD166). We found that the Li-7 cell line exhibited a “population change” from CD13(+)/CD166(−) slow-glowing CSCs to CD13(−)/CD166(+) rapidly-growing progenitor cells. The effects of sorafenib and 5-fluorouracil (5-FU) were then tested in this model cell line: sorafenib and 5-FU were found to selectively target CSCs and progenitor populations, respectively. We also found that a sequential combination of the two drugs (5-FU followed by sorafenib) produced more potent cytotoxic effects than the reverse sequence or either alone. Li-7 is therefore a valuable cell line to study the mechanisms of CSC differentiation and chemoresistance, and to explore drugs targeting CSCs *in vitro* in order to develop better therapies for HCC.

## Methods

### Cell lines

The human HCC cell lines HuH-7 [21] and Li-7 [22] were provided by RIKEN BRC through the National Bio-Resource Project of MEXT (RIKEN cell bank, Tsukuba, Japan); the other human HCC cell lines, PLC/PRF/5 [23], HLE and HLF [24], were provided by the Japanese Collection of Research Bioresources Cell Bank (JCRB cell bank, Osaka, Japan). HuH-7, Li-7 and PLC/PRF/5 cells were maintained in RPMI 1640 supplemented with 10% fetal bovine serum (FBS). HLE and HLF cells were maintained in DMEM supplemented with 10% and 5% FBS, respectively. All cells were cultured at 37°C with 5% partial pressure of CO_2_ in a humidified atmosphere. Cells were passaged twice a week in 10 cm diameter tissue culture dishes, usually at approximately 80% confluency, without medium exchange.

### Flow cytometric analysis

Cells (5 × 10^5^) were labeled with the following human antibodies: phycoerythrin (PE)-conjugated CD166 (ALCAM; BD Bioscience, San Jose, CA), CD324 (EpCAM; eBioscience, San Diego, CA), CD133 (Miltenyi Biotec, Bergisch Gladbach, German), CD44 (eBioscience), fluorescein isothiocyanate (FITC)-conjugated CD44 (eBioscience), biotin-conjugated CD24 (eBioscience), CD133 (Miltenyi Biotec), allophycocyanin (APC)-conjugated CD13 (eBioscience), CD133 (Miltenyi Biotec), and CD90 (eBioscience). The following isotype-matched mouse or rat immunoglobulins were used as controls: APC-conjugated mouse IgG1 (BD biosciences), mouse IgG2b (eBioscience), PE-conjugated mouse IgG1 (R&D Systems Inc., Minneapolis, MN), FITC-conjugated rat IgG2b (R&D Systems Inc.), biotin-conjugated mouse IgG1 (R&D Systems Inc.). Cell samples were analyzed by flow cytometry using a FACSCalibur (BD biosciences) and CellQuest software (Version 6.0, BD biosciences). 7-AAD (BD biosciences) was used to identify dead cells.

### Cell sorting

Cells were labeled with fluorescent dye-conjugated antibodies and sorted by flow cytometry using a FACSAria II (BD biosciences) and FACSDiva software version 6.1 (BD biosciences). Doublet cells were eliminated using FSC-H and FSC-W, SSC-H and SSC-W. Dead cells were eliminated as 7-AAD-positive cells. For the positive and negative populations, the top 25% of intensely stained cells or the bottom 20% of unstained cells were selected to be sorted, respectively. Post-sort analysis was performed to confirm that purity of cell fractions was more than 90%.

### Cell proliferation and chemosensitivity assay

For the cell proliferation assay, cells were seeded into 96-well plates at 3 × 10^3^ cells per well and cell viability was measured at 24, 48, 72 and 96 hr after sorting using the Cell Counting Kit-8 (Dojindo, Kumamoto, Japan). Absorbance was detected by a 2030 Multilabel Reader (ARVO X3; PerkinElmer, Waltham, MA). For the chemosensitivity assay, cells were seeded into 96-well plates at 3 × 10^3^ cells per well and 5-FU (Kyowa Hakko Kirin, Tokyo, Japan) or sorafenib tosilate (Bayer Healthcare Pharmaceuticals, Osaka, Japan) was added; cell viability was measured 72 hr later. Sorafenib was dissolved in DMSO at 10 mM and further diluted in fresh medium [25]. Bulk cells of several cell lines were seeded into 96-well-plate at 5 × 10^3^ cells per well and incubated overnight at 37°C. The medium was then replaced by medium containing different concentrations of sorafenib tosilate. Cell viability at 72 h was measured in the same manner as in the proliferation assay.

### Aldefluor assay

ALDEFLUOR reagent (Stemcell Technologies, Vancouver, BC, Canada) was used for the detection of intracellular ALDH1 enzymatic activity [16]. The assay was performed according to the manufacturer’s instructions. Briefly, 0.12 μg/mL BODIPY- aminoacetaldehyde (BAAA), a fluorescent substrate for ALDH, was added to 5 × 10^5^ cells, which were then incubated at 37°C in a water bath for 10 mins. For the negative control, 15 μM diethylaminobenzaldehyde (DEAB), a specific inhibitor of ALDH, was added to the reaction cocktail. After incubation, samples were centrifuged to collect cells, which were then stained with fluorescent dye-conjugated anti-CD13 and anti-CD166 antibodies. Immunofluorescent detection was performed with a FACSAria II (BD Biosciences) using a yellow-green laser for PE conjugated CD166, a blue laser for Aldefluor and 7-AAD, and a red laser for APC conjugated CD13. The data analysis was carried out using FloJo software (Version 7.6, Tomy Digital Biology, Tokyo, Japan).

### Spheroid colony assay

Sorted cells were seeded at 3 × 10^3^ cells per well into a 96-well Nanoculture plate (NCP)-MS (Scivax, Kawasaki, Kanagawa, Japan) with 150 μl of NanoCulture medium R type supplemented with 10% FBS-R (Scivax). Half of the medium volume was replaced every 3 to 4 days. Spheroid colonies with a diameter in excess of 100 μm were counted on day 20 using a microscope equipped with a digital camera (DP25, Olympus, Tokyo, Japan) in combination with imaging software (CellSens, Olympus). Li-7 cells were seeded at 1×10^3^ cells per well in 3 wells of 96-well NCP-MS as described above. Spheroid colonies were dispersed using spheroid dispersion solution (Scivax) and seeded in a well of a 24-well NCP-MS plate (Scivax) on day 14. Spheroid colonies were then harvested on day 24 and analyzed by flow cytometry.

### Immunocytochemistry

Li-7 cells were seeded on a chamber slide (LAB-TEK, Hatfield, PA) at 1 × 10^4^ cells per well with 0.5 ml of medium. On day 10, the cells were fixed with 4% paraformaldehyde for 15 min and incubated with anti-CD166 mouse monoclonal antibody (BD Biosciences), and anti-Ki-67 rabbit polyclonal antibody (abcam, Cambridge, MA) at 4°C overnight. The cells were stained with secondary antibodies using goat anti-rabbit IgG conjugated with Alexa Fluor 546 (Life technologies, Carlsbad, CA) and goat anti-rabbit IgG conjugated with Alexa Fluor 488 (Life technologies) at room temperature for 1 hr. Cells were mounted with mounting solution with DAPI (Vector, Olean, NY) and covered with a coverslip (Matsunami, Osaka, Japan). A BX51 fluorescence microscope (Olympus) and imaging software (cellSens; Olympus) was used to analyze fluorescence digital images.

### Microarray analysis

CD166(−) and CD166 (+) cells were sorted from bulk Li-7 cells and total RNAs were extracted using an RNeasy kit (Qiagen, Valencia, CA). Samples of RNA were quantified with a spectrophotometer and then used to generate Cy-3-labelled cRNA according to the manufacturer’s instructions. The dye content and concentration of cRNA were measured by spectrophotometry (NanoDrop Technologies, Wilmington, DE). A 1650 ng aliquot of Cy3-labelled cRNA was hybridized to oligonucleotides immobilized on the surface of microarray slides (Agilent Technologies, Palo Alto, CA) at 65°C for 17 hr; the slides were washed and treated with Gene Expression Wash Buffer (Agilent Technologies) and then scanned using an Agilent Microarray Scanner. All steps were performed according to the manufacturer’s instructions (Agilent Technologies). The data was analyzed with GeneSpring software (Version 12.5, Agilent Technologies).

### Animal experiments

Three to four-week-old female BALB/c *nu/nu* nude mice were purchased from CLEA Japan, Inc (Tokyo, Japan). Bulk Li-7 cells (1 × 10^6^ cells), which had been passaged at different times, were injected subcutaneously at 2 sites into each mouse. The mice were sacrificed after apparent subcutaneous tumors were observed or at 4 months after injection. All animal experiments were approved by the Institutional Animal Care and Use Committee of RIKEN BioResource Center (14–003).

### Immunohistochemistry and flow cytometry of a xenograft tumor

A half of a xenograft tumor was cut into pieces, placed into RPMI supplemented with 5% FBS with 2 mg/ml collagenase mixture and incubated for 30 min at 37°C. Cells were filtered through a 40 μm cell strainer (BD Biosciences, Bedford, MA) and stained with antibodies. Dead cells and doublet cells were eliminated as described above. The remaining part of the xenograft tumor was fixed with 4% paraformaldehyde and then paraffin embedded. Immunohistochemical staining was performed using mouse anti-human CD13 monoclonal antibody (eBioscience) and rabbit anti-human Ki-67 polyclonal antibody (Abcam).

### Statistics

Fisher’s exact test was used to identify significant differences in tumorigenicity. Student’s *t*-test was employed to identify significant differences in cell proliferation rates and chemosensitivity. A value of *P* < 0.05 was considered significant. SPSS V22 (IBM Japan, Tokyo) software was used for all statistical analyses.

## Results

### Population change in HCC cell lines

**Figure 1:**
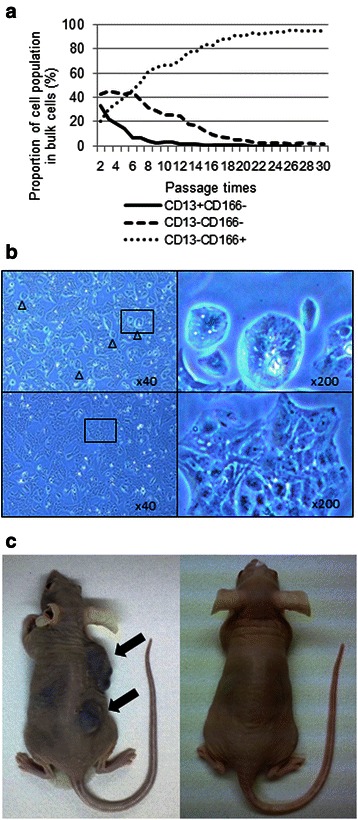
Changes in subpopulations of Li-7 cells during culture and effects on cell morphology and tumorigenicity. **a)** Analysis of CD13 and CD166 expression in Li-7 cells during culture by flow cytometry. The proportion of cells expressing CD13 decreased and that of cells expressing CD166 increased with the number of passages. **b)** Morphological changes in the bulk Li-7 cells after 2 months in culture. Upper panel, Li-7 cells after one week of culture; lower panel, Li-7 cells after 2 months of culture. **c)** Injection of Li-7 cells (1 × 10^6^) after one week of culture into nude mice caused tumor formation in all mice after 2 months, whereas the cells injected after 2 months of culture were non-tumorigenic even at 4 months (right).

In order to identify HCC cell lines with a preserved CSC hierarchy, we screened cell populations for changes in the expression of various cell surface markers (population change). We used the markers CD13, EpCAM, CD133, CD44, CD90, CD24 and CD166 and screened HuH-7, Li-7, PLC/PRF/5, HLF, and HLE cell lines by FACS before and after culture for 2 months. Only the Li-7 cell line showed a population change: the FACS analysis indicated that in this cell line the proportion of CD13(+) cells decreased, while that of CD166(+) cells increased after 2 months in culture (Table 1). We confirmed this change by examining expression of the markers by FACS analysis after each passage. This analysis demonstrated that the CD13(+)/CD166(−) population disappeared within 1 month. By contrast, the CD13(−)/CD166(+) population gradually increased and became dominant in the bulk Li-7 cells after 2 months (Figure 1a). This pattern was found consistently in independent experiments. We tested whether the relative sizes of the subpopulations after the 2 months culture reverted to the initial state following freezing and thawing of the cells. The proportions of the two markers were unchanged after freezing/thawing, suggesting that the change during the short culture period was irreversible. The altered patterns of marker expression were accompanied by changes in the morphological appearance of the Li-7 cells. Small clusters of round cells were observed at the initial culture stages (within a few passages), but decreased in numbers with time in culture (after several passages) and were very infrequent after 2 months of culture (Figure 1b). These morphological changes supported our interpretation of a population change in Li-7 cells.

**Table 1:** **CSC markers detected by flow cytometry in HCC cell lines before and after 2 months culture**

	Li-7		HuH-7		PLC/PRF/5		HLF		HLE	
	pre	post	pre	post	pre	post	pre	post	pre	post
**CD133**	**+**	**+**	**++**	**++**	**-**	**-**	**-**	**-**	**-**	**-**
**EpCAM**	**++**	**++**	**++**	**++**	**-**	**-**	**-**	**-**	**-**	**-**
**CD90**	**-**	**-**	**-**	**-**	**+−**	**+−**	**+−**	**+−**	**++**	**++**
**CD24**	**++**	**++**	**++**	**++**	**++**	**++**	**++**	**+**	**++**	**++**
**CD44**	**++**	**++**	**+**	**+**	**+−**	**+−**	**++**	**++**	**++**	**++**
**CD13**	**+**	**-**	**++**	**++**	**++**	**++**	**-**	**-**	**-**	**-**
**CD166**	**+**	**++**	**++**	**++**	**++**	**++**	**++**	**++**	**++**	**++**

pre, culture for one week; post, culture for 2 months.

-, less than 5%; +−, 5 to 30%; +, 30 to 70%; ++, more than 70%.

We compared the tumorigenicity of Li-7 cells before and after the population change. After one week in culture, CD13(+)/CD166(−) cells comprised about 20% of Li-7 cells: injection of these bulk Li-7 cells resulted in subcutaneous tumors at every injection site (4/4; Figure 1c, Table 2). After one month of culture, the Li-7 cells had no CD13(+)/CD166(−) cells but contained only CD13(−)/CD166(−) and the CD13(−)/CD166(+) cells: injection of these Li-7 cells resulted in the formation of a tumor at only one of 4 sites at 2 months after injection. After 2 months of Li-7 cell culture, the population mostly comprised CD13(−)/CD166(+) cells, and no tumors had formed even at 4 months post-injection in nude mice (0/4; Figure 1c, Table 2). Therefore, the high tumorigenicity of Li-7 cells in nude mice was completely lost during the culture period when a population change occurred.

**Table 2:** **Loss of tumorigenicity of Li-7 cells according to passage times**

Culture period	Tumorigenicity
1 week (containing CD13+/CD166-)	4/4
4 weeks (CD13-/CD166- and CD13-/CD166+)	1/4
8 weeks (only CD13-/CD166+)	0/4*

*p = 0.01.

### *In vitro* hierarchy of Li-7 cells

**Figure 2:**
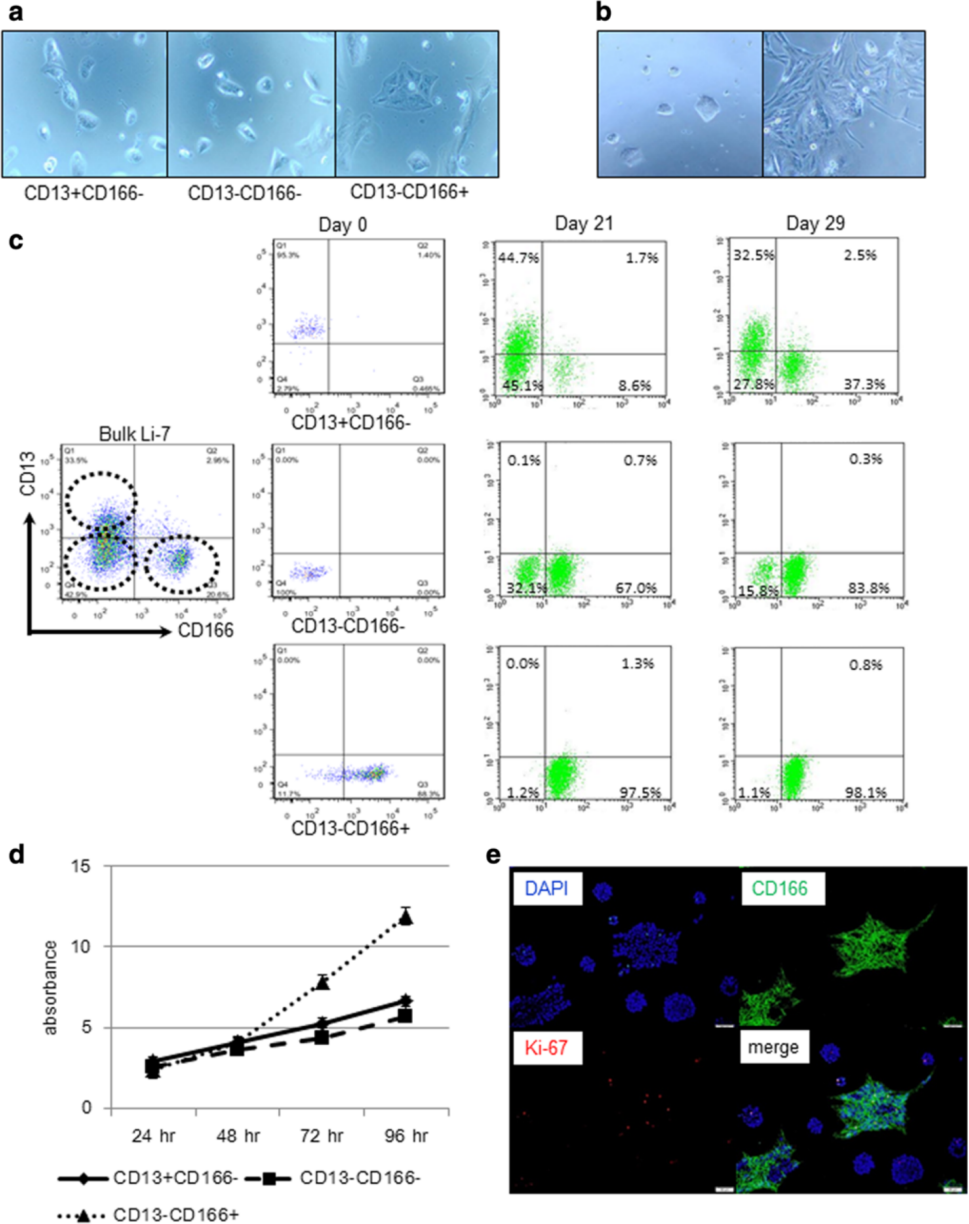
Slow-growing CD13(+)/CD166(−) cells could reconstruct the bulk Li-7 cell population. **(a)** Microscopic appearance of cell subpopulations at 72 hr after cell sorting from Li-7 cell culture: CD13(+)/CD166(−) and CD13(−)/CD166(−) cells grew slowly as round cell clusters, whereas CD13(−)/CD166(+) cells attach strongly to the dish and grew rapidly. **(b)** CD166(−) cell clusters elongated and spread as CD166(+) cells while increasing the number of cells in a cluster. **(c)** Expression of CD13 and CD166 in cell fractions sorted from Li-7 cultures and subcultured for different periods. CD13(+)/CD166(−) cells produced CD13(−)/CD166(−) and CD13(−)/CD166(+) cells and were able to reform the bulk Li-7 cell population (upper). CD13(−)/CD166(−) cells only produced CD13(−)/CD166(+) cells (middle). The CD13(−)/CD166(+) cells did not produce other fractions (lower). **(d)** Cell growth rates of each subpopulation using WST-8 showing that CD13(−)/CD166(+) cells grew rapidly compared to CD166(−) cells. **(e)** Immunocytochemical staining of bulk Li-7 cells revealed widespread expression of Ki-67 in CD166(+) cell colonies.

We next investigated whether the Li-7 cells were composed of hierarchically heterogeneous cell populations in which CD13(+)/CD166(−) cells formed the CSC population and CD13(−)/CD166(+) cells formed the progenitor population. We separately fractionated the three types of cells using marker expression patterns and then analyzed whether the isolated cells populations could generate other population(s). Most of the CD13(+)/CD166(−) cells grew as clusters of round cells, resembling some of the cells in bulk Li-7 culture (Figure 2a). The number of cells in a cluster increased and the clusters elongated and spread (Figure 2b). FACS analysis showed that the CD13(+)/CD166(−) cells produced a CD13(−)/CD166(−) population within 3 weeks. After one month of subculture following FACS sorting, the proportion of CD13(−)/CD166(+) cells increased to approximately 40%. In association with these changes in marker expression, round cell clusters gradually diminished in number. On the other hand, the CD13(−)/CD166(−) cells produced CD13(−)/CD166(+) cells but no CD13(+)/CD166(−) cells. We found that CD13(−)/CD166(+) cells did not produce any other types of cell during a one month culture period (Figure 2c). From these results, we conclude that only the CD13(+)/CD166(−) cells have the ability to produce the range of cell types in the Li-7 cell populations and, thus, that they must be superior to other cell types in the hierarchy of Li-7 cells.

During the culture of Li-7 subpopulations, we noticed that the CD13(−)/CD166(+) cells grew faster than CD166(−) cells. To confirm this impression, we compared the cell growth characteristics of each subpopulation of Li-7. We found that CD13(−)/CD166(+) cells grew considerably faster than CD166(−) cells, and that CD13(+)/CD166(−) cells grew equally slowly as CD13(−)/CD166(−) cells until 96 hr after sorting (Figure 2d). We set up cultures with low concentrations of bulk Li-7 cells to ensure that each cell colony grew separately and analyzed the cultures for Ki-67 staining. We found that Ki-67 was expressed mainly in CD166(+) cell colonies, thus confirming that these cells were the rapidly growing progenitor cells in the Li-7 cell line (Figure 2e).

### Functional hierarchy in Li-7 cells

**Figure 3:**
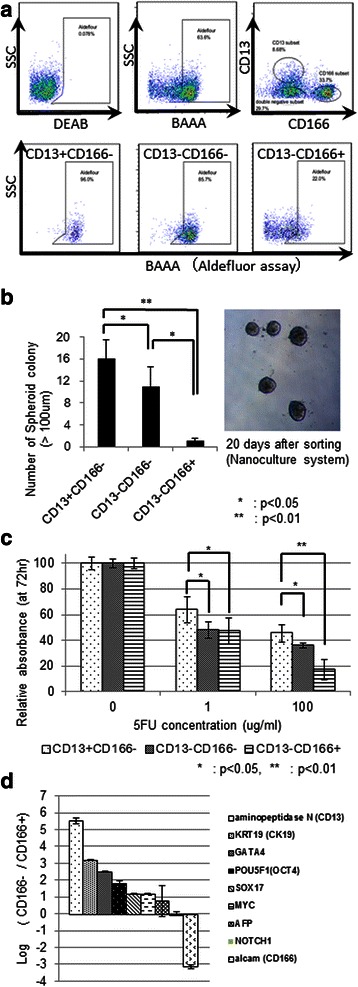
Functional hierarchy in Li-7 cells *in vitro*. **a)** Flow cytometry of cells prepared for an Aldefluor assay and immunostained for CD13 and CD166. Upper panels: Aldefluor assay of the bulk Li-7 cell population (left: BAAA with DEAB, middle: only BAAA) and CD13 and CD166 (right). Lower panels: Aldefluor assay of gated fractions (left: CD13(+)/CD166(−), middle: CD13(−)/CD166(−), right: CD13(−)/CD166(+), showing the highest and the lowest ALDH activities in the CD13(+)/CD166(−) and CD13(−)/CD166(+) cells, respectively. **b)** Spheroid colony assay showed a high ability of CD13(+)/CD166(−) cells and a relatively low ability of CD13(−)/CD166(+) cells to produce colonies. **c)** 5-FU treatment of the subfractions showed a relatively higher sensitivity of CD13(−)/CD166(+) cells and lower sensitivity of CD13(+)/CD166(−) cells (72 hr; WST-8 assay). **d)** A microarray analysis showed relatively higher levels of expression of stemness-related genes in CD166(−) cells than CD166(+) cells.

To investigate functional hierarchies in the Li-7 cell line, we performed an Aldefluor assay in combination with double staining for CD13 and CD166. This analysis showed that most (96%) CD13(+)/CD166(−) cells had a high level of ALDH activity (Figure 3a). A large proportion (85.7%) of CD13(−)/CD166(−) cells also showed high ALDH activity. By contrast, only 22% of CD13(−)/CD166(+) cells showed ALDH activity (Figure 3a). The analysis therefore demonstrated that the CD13(+)/CD166(−) cells retained one of the critical features of CSCs [17].

We next examined the Li-7 cell cultures for spheroid formation, another characteristic of CSC [26]. We sorted each fraction and directly plated the subpopulations onto low-attachment plates. The CD13(+)/CD166(−) cells formed many large spheroid colonies, particularly in comparison to CD13(−)/CD166(−) cells. The CD13(−)/CD166(+) cells had the lowest ability to form spheroid colonies among the three fractions (Figure 3b). We examined spheroid formation in bulk Li-7 cells and confirmed that it decreased after the population change. Interestingly, most cells in the spheroid colonies produced by bulk Li-7 cells expressed CD13 but not CD166 (Additional file 1: Figure S1). Cells in spheroid colonies from CD13(+)/CD166(−) cells or even from CD13(−)/CD166(−) cells also mostly expressed CD13 (Additional file 1: Figure S1), although CD13 expression decreased after subculture under normal conditions.

We examined the response of the Li-7 cells to 5-FU treatment and found that growth of CD13(−)/CD166(+) cells was preferentially suppressed, whereas that of CD13(+)/CD166(−) cells was affected least (Figure 3c). We also found that bulk Li-7 cells became relatively more sensitive to 5-FU after the population change (data not shown).

Finally, we performed a microarray analysis to compare expression of stemness-related genes in CD166(+) and CD166(−) cells. The analysis revealed that several stemness-related genes, including *OCT4*, *SOX17* and *MYC* [13,14,16], were expressed at higher levels in CD166(−) cells than in CD166(+) cells (Figure 3d). In addition, the mRNA levels of *KRT19*, which is considered to be immature marker in HCC [9,10], were higher in CD166(−) cells than in CD166(+) cells.

### CD13 expression *in vivo*

**Figure 4:**
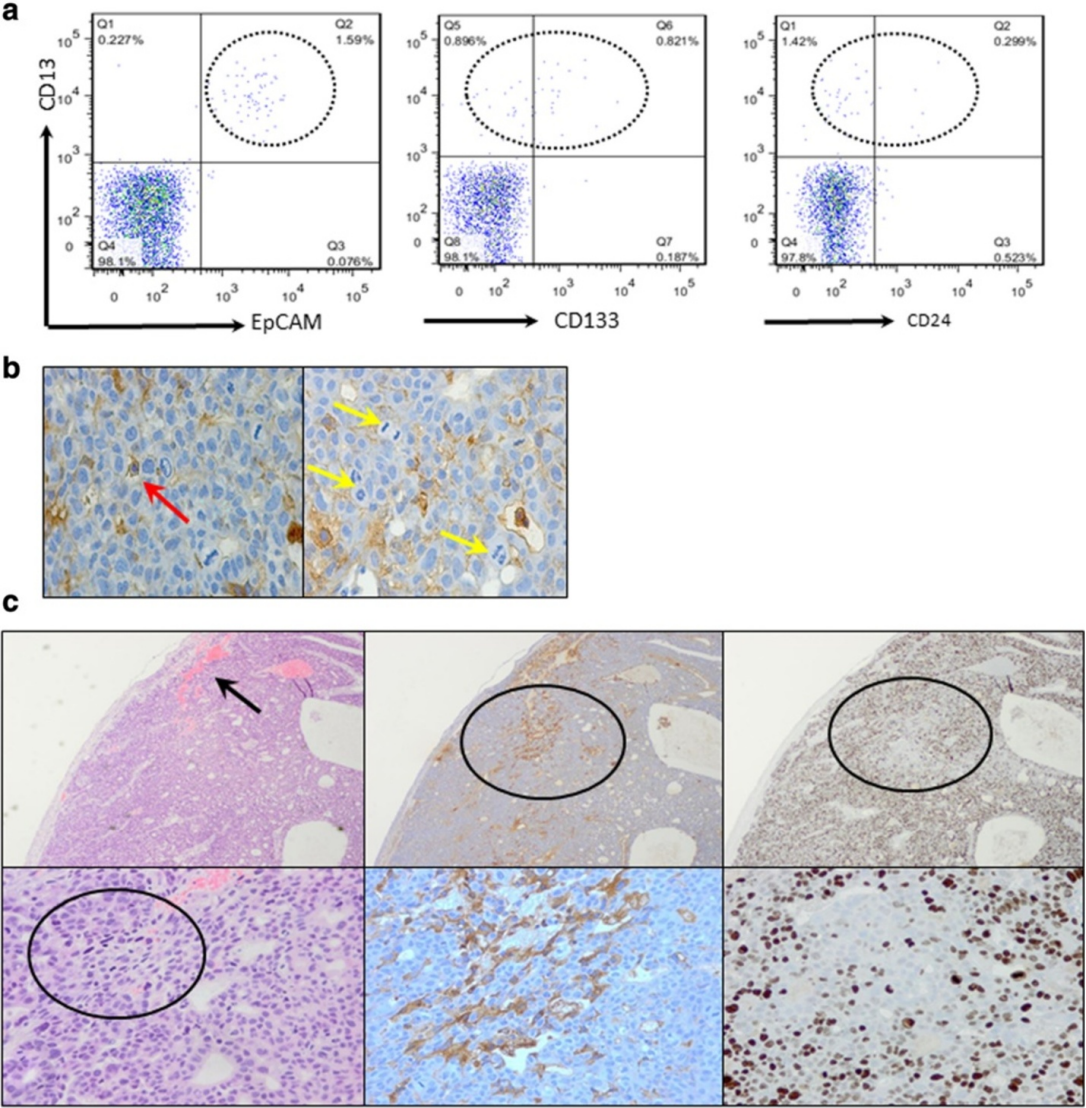
CD13 is a marker for slow-growing CSCs *in vivo*. **a)** FACS analysis of a xenograft tumor produced by Li-7 cells showed an association of CD13 expression with other CSC markers (left: EpCAM, middle: CD133, right: CD24). **b)** Immunohistochemical localization of CD13(+) cells in xenograft tumors. Some parts of the tumor stained (red arrow) but cells in mitosis were unstained (yellow arrow). **c)** Ki-67 (right) and CD13 (middle) expression and hematoxylin and eosin staining (left) of a xenograft tumor showed absence of Ki-67 staining in morphologically undifferentiated CD13(+) cells near the vessels (black arrow).

We examined whether CD13 might serve as a marker for slow-growing CSCs *in vivo*. First, we performed a FACS analysis of xenograft tumor tissues in nude mice that resulted from the injection of bulk Li-7 cells. Double staining of cells for CD13 and EpCAM, CD133 or CD24 revealed that these other CSC markers were co-expressed with CD13 (Figure 4a). Although EpCAM, CD133, CD24 and CD44 were expressed in all three subpopulations of Li-7 cells *in vitro* (Additional file 2: Figure S2), interestingly, they were expressed only in a very low proportion of tumor cells expressing CD13 *in vivo*. The data suggested that there were differences in the expression patterns of CSC markers *in vitro* and *in vivo*, and that CD13 in Li-7 cells might serve as a CSC marker both *in vitro* and *in vivo*.

We also performed an immunohistochemical analysis of the same xenograft tumor to analyze the distribution of CD13 and Ki-67 expressing cells. CD13 was only expressed by a few tumor cells, and was not present in mitotically active cells (Figure 4b). Focal expression of CD13 was identified in a lesion near a vessel: hematoxylin-eosin staining of the cells involved showed them to be small with dense nuclear chromatin and a high nuclear-cytoplasmic ratio, features compatible with undifferentiated cells (Figure 4c). Ki-67 expression was low in these cells. These findings suggest that CD13 expression was present in morphologically undifferentiated slow-growing CSCs *in vivo*.

### Effects of treatment with sorafenib and/or 5-FU

**Figure 5:**
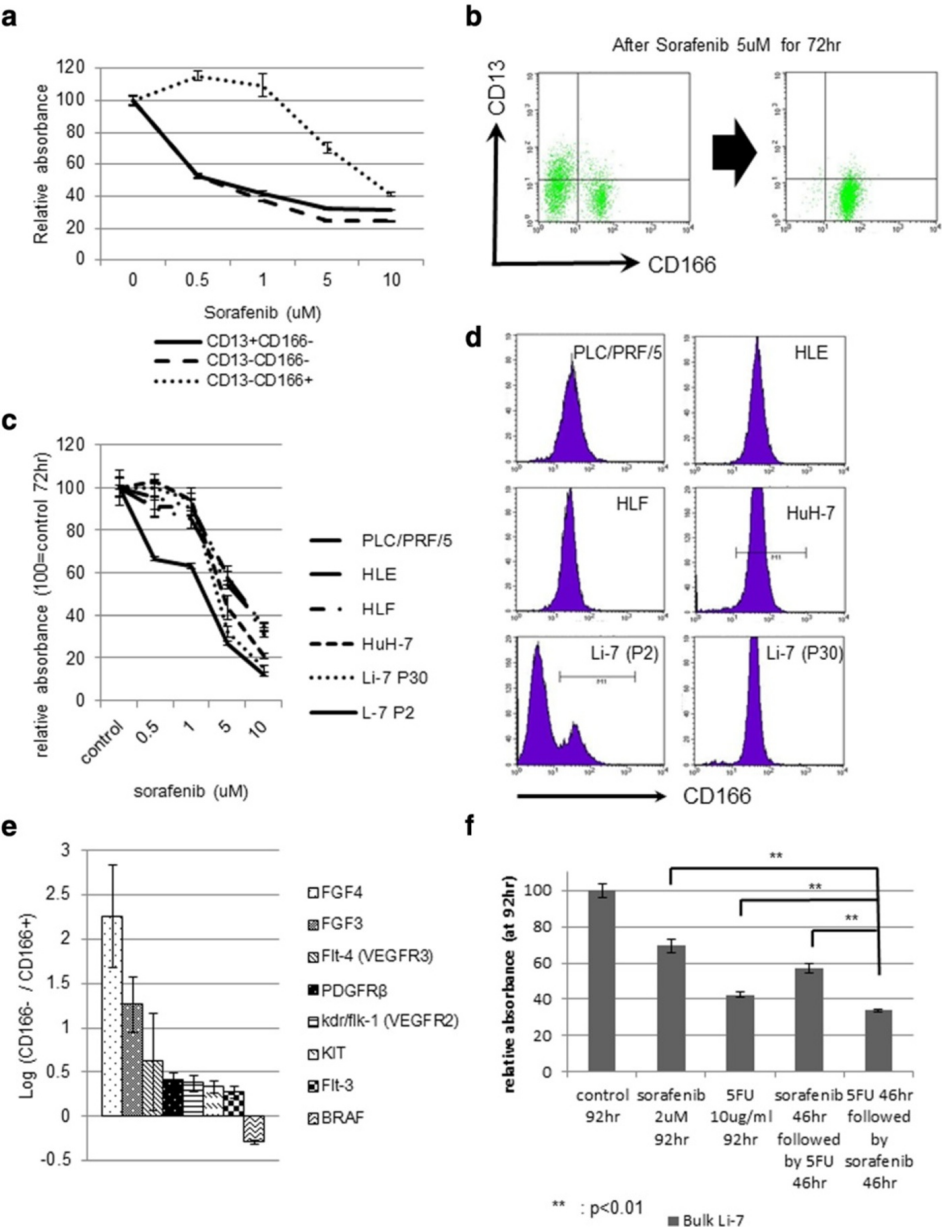
Sensitivities of Li-7 subpopulations to sorafenib. **a)** Sorafenib treatment (72 hr) selectively killed CD166(−) cells but not CD166(+) cells (WST-8 assay). **b)** FACS analysis of the bulk Li-7 cell population before (left) and after (right) sorafenib treatment (5 μM, 72 hr) confirmed selective killing of CD166(−) cells by sorafenib. **c)** Li-7 cells were more sensitive to sorafenib than other cell lines or Li-7 cells that have undergone 30 passages (WST-8). **d)** HCC lines other than Li-7 showed high expression of CD166 (FACS), which explains their resistance to sorafenib. **e)** Microarray analysis showed that *FGF3*, *FGF4* and sorafenib-targeted genes are more highly expressed in CD166(−) cells than CD166(+) cells. **f)** Sequential treatment with 5-FU followed by sorafenib more effectively suppressed growth of Li-7 cells (*P* <0.01) than either alone (WST-8).

Next, we examined the effect of sorafenib on Li-7 cell subpopulations. Sorafenib selectively killed CD166(−) but not CD166(+) cells (Figure 5a). In addition, when sorafenib (5 μM) was added to bulk Li-7 cells for 72 h, only CD166(+) cells survived, confirming the selective killing of CD166(−) cells by sorafenib (Figure 5b). The bulk Li-7 cells showed greater sensitivity to sorafenib compared with other cell lines (HLE, HLF, PLC/PRF/5, HuH-7) that express high levels of CD166 (Figure 5c,d). Thus, CD166 might be a marker associated with resistance to sorafenib. CD13(+)/CD166(−) and CD13(−)/CD166(−) cells showed similar sensitivities to cell killing by sorafenib. We performed a microarray analysis in CD166(−) and CD166(+) cells to compare the expression of genes targeted by sorafenib. Several genes, including *VEGFR*, *PDGFR* and *Flt*-3 (but not *BRAF*) were expressed at a higher level in CD166(−) cells compared with CD166(+) cells. In addition, expression of *FGF3* and *FGF4*, which has been observed to show amplification only in sorafenib responders [27], were also significantly higher in CD166(−) fraction (Figure 5e). These observations support the conclusion that the CD166(−) fractions, including CD13(+)/CD166(−) CSCs, are the target of sorafenib.

By contrast to the results of sorafenib treatment, 5-FU preferentially suppressed the growth of CD166(+) cells (Figure 3c). Thus, we also examined whether sorafenib would work more efficiently in combination with 5-FU. We found that 5-FU followed by sorafenib suppressed the growth of bulk Li-7 cells more efficiently than either alone (Figure 5f). Additionally, the combination of the two drugs in this order was more effective than sorafenib followed by 5FU (Figure 5f).

## Discussion

Recent CSC research has proved that many cell lines contain a cell subpopulation with a CSC phenotype and are thus “heterogeneous”. We examined here, for the first time, whether several HCC cell lines are “stable” or “unstable” during culture for 2 months. We demonstrated that only the Li-7 cell line of the tested HCC cell lines showed a “population change”(phenotypic changes during culture) in the expression pattern of cell surface markers, cell appearance, and tumorigenicity surprisingly. We also found that the Li-7 cell line is composed of hierarchically heterogeneous cell populations with CD13(+)/CD166(−) cells acting as slow-growing CSCs and CD13(−)/CD166(+) cells acting as rapidly-growing progenitor cells (Figure 2d and e, Additional file 3: Figure S3). In addition, we demonstrated that the differentiation of the CSCs into the progenitor cells (Figure 2a-c) and the differences in the growth rates of the two subpopulations (Figure 2d and e) were responsible for the population change. To our knowledge, this is the first identification of a cancer cell line that undergoes a “population change” due to the differentiation of CSCs during culture. We also showed that sorafenib and 5-FU preferentially targeted CSC and progenitor populations, respectively (Figure 3c, Figure 5a), and that sequential treatment with the two drugs had potent cytotoxic activity in the Li-7 cell line (Figure 5f). Thus, the Li-7 cell line is highly heterogeneous and will be of value in studying the mechanisms of CSC differentiation and chemoresistance and is also useful for investigating candidate drugs that target the CSC *in vitro* to identify new therapeutic strategies for HCC. Cell lines with similar characteristics to Li-7 cells might prove useful for cancer research in the CSC era and important for cell banks to meet various demands of researchers.

The most probable reason why Li-7 shows this “population change” is that it is a unique cell line that was originally developed following *in vivo* transplantation in mice and maintained similarly by Hirohashi, et al. [22]; the cells were deposited into our RIKEN cell bank shortly after development. Therefore, Li-7 retained its initial “heterogeneous” character *in vitro* in contrast to other cell lines that were established after extensive cultures *in vitro* [21,23,24]. The results from the culture experiments here suggest that if the Li-7 cells had originally been cultured *in vitro* for a longer period before being deposited into the cell bank, then the cell line would likely have comprised only CD13(−)CD166(+) cells with no CD13(+) CSC cells present; thus it would have become “stable” like other cell lines. Although “heterogeneous and unstable” cell lines have been considered as unsuitable research materials in the past, our findings here suggest that these types of cells might better be considered as “new major players” in the CSC era. It may be useful for cancer research to establish more cell lines with similar characteristics to Li-7 cells by the way this cell line was established. We also found that HepG2, one of the most popular hepatoblastoma cell lines [28], also demonstrates a population change in which the proportion of cells expressing CD13 decreased, while that of cells expressing CD133 increased during culture (data not shown). This population change might explain the reported variation in CD133 expression rates between 0.28% and 41% for HepG2 cells [11,12,29,30].

Apart from Li-7 cells, the HCC cell lines tested were relatively “homogeneous” in terms of the expression pattern of CD13 and CD166 than Li-7 cells, and only contained either CD13(+) CD166(+) (in HuH-7 and PLC/PRF/5 cells) or CD13(−)CD166(+) population (in HLE and HLF cells); they also displayed a “stable” population pattern during culture *in vitro* (Table 1). The most probable reason for this is that they were highly clonogenic cells due to having been extensively cultured *in vitro* before deposition in a cell bank. It is also possible that differentiation of CSCs was arrested in some cell lines for some reason. When these cell lines are transplanted *in vivo,* CSCs might possibly emerge because of the “plasticity” [31] of the cells and exert their functions according to the *in vivo* “niche” [32]. This may explain why CSCs have been identified in these cell lines, even though their CSC functions could not be clearly determined *in vitro*. Further detailed studies are necessary to clarify the basis of the differences between the cell lines that undergo a “population change” and those that are “stable” *in vitro*.

CD13 was found to be a semi-quiescent CSC marker for HCC by Haraguchi, et al. [18] They also demonstrated the efficacy of a CD13 (aminopeptidase N) inhibitor, Bestatin, both *in vitro* and *in vivo* [18]; this drug has been approved for the maintenance treatment for acute myeloid leukemia in Japan. Martin-Padura et al. have reported that CD13(+) cells are responsible for tumor relapse in a xenograft model [33]. However, these studies have not demonstrated a simple *in vitro* model which can monitor the differentiation of CD13(+) CSC*.* Here, we confirmed that CD13(+) cells are slow-growing CSCs in the Li-7 cell line by several *in vitro* functional assays. In addition, we extended the results of the earlier studies [18,33] by identifying Li-7 cells as an HCC line capable of tracing the differentiation of CD13(+) CSCs and for testing the effects of drugs on CD13(+) CSCs.

Other CSC markers, including EpCAM, CD133, CD24 and CD44, were expressed in all three subpopulations of Li-7 cells (Additional file 2: Figure S2), indicating that these markers did not selectively recognize slow-growing CSCs but also recognized rapidly-growing progenitor cells *in vitro*. Indeed, most of these markers have not been reported as markers of “slow-growing” CSCs [12,15,16]. Furthermore, some reports have suggested that a CSC marker in one cell line may not necessarily be a CSC marker in other cell lines [34]. Thus, each cell line may have a unique set of CSCs marker *in vitro*. Very interestingly, however, these markers were expressed only in CD13(+) cells *in vivo* (Figure 4a). There are various possible reasons for the different patterns of expression of CSC markers *in vitro* and *in vivo*. First, the regulation of CSCs is influenced by the tumor microenvironment or niche [32]. Indeed, we found that CD13(+) cells focally accumulated near vessels in a xenograft tumor developed by Li-7 cell injection (Figure 4c). Second, there may be plasticity with regard to dormancy [1,31] or in the reprogramming from progenitors to CSCs [35]. We found that CD13(−)/CD166(−) cells grown under normal culture conditions expressed CD13 in spheroid colonies (Additional file 1: Figure S1). This strongly supports the notion of plasticity of CD13 expression and suggests that culture conditions promoting spheroid formation might be suitable for suppressing the population change and maintaining CD13(+) CSCs. CSC models are usually much more complicated *in vivo* than *in vitro* [36,37]. However, the Li-7 cells seemed to maintain their complex character *in vitro*, including high “heterogenicity” and “instability”(due to the CSC differentiation) compared to other HCC cell lines tested. As discussed above, this characteristic of Li-7 cells may be due to its culture history before cell bank deposition [22].

Several other approaches to label and trace CSCs *in vitro* have been proposed by other investigators, but these methods may be limited by their requirement for specific genetic labeling [19,38,39]. By contrast, our Li-7 cell line offers a simple approach that does not entail genetic marking of the CSCs. Moreover, the cell line is freely available to all researchers from a public cell repository, although periodic re-cloning to select CD13(+) cells is required to maintain the precious characteristics of the Li-7 cells. The Li-7 cell line enables identification of both a slow-growing CSC fraction and a rapidly-growing progenitor fraction using CD13 and CD166 markers, respectively, and this allows this cell line to be useful for screening drugs that target CSCs or progenitors *in vitro*. Few other model systems permit simultaneous identification of slow-growing CSCs and progenitor cells. This characteristic enables this cell line to be of use for screening drugs that target CSCs or progenitors *in vitro.*

We confirmed that 5-FU, one of the most widely-used cytotoxic agents, preferentially targets CD166 (+) progenitor cells. Previous reports have demonstrated that CSCs are relatively resistant to conventional chemotherapy because they express transporters, including ATP-binding cassette transporters, to excrete drugs [40] and to reduce ROS levels to avoid DNA damage after chemotherapy [18].

Sorafenib more effectively killed CD166(−) cells than CD166(+) cells in Li-7 cultures (Figure 5a, b). It was previously reported that the cell subpopulation with high ALDH activity in the HLE cell line is sensitive to sorafenib [41] and that sorafenib can target CSCs together with a PI3K inhibitor [42]. Other reports have suggested that the CSCs in HCC are resistant to sorafenib [38,39]; however, the CSCs in these studies were not slow-growing but rather formed a rapidly-growing subpopulation. Selective targeting of CSCs by sorafenib would be a reasonable expectation since the drug inhibits VEGF signals, which have recently been postulated to play an important role in CSC self-renewal [43]. In addition, WNT/beta-catenin signaling, which cross-talks with FGF signaling, plays a very important role in CSC maintenance [44]. Clinically, amplification of FGF3 and FGF4 has been observed only in sorafenib responders [27]. We found significantly higher expression of *FGF3* and *FGF4* genes in the CD166(−) fraction (Figure 5e), supporting interpretation that sorafenib affects the CD166(−) fraction through the FGF and wnt/beta-catenin signaling pathways.

We found stronger growth inhibition of Li-7 cells by sequential treatment with 5-FU followed by sorafenib than with either alone (Figure 5f). Previous studies have shown that sorafenib induces G0 cell cycle arrest and impairs the cytotoxicity of 5-FU [45] or radiotherapy [46]. Indeed, the reverse order of sorafenib followed by 5-FU did not show significant effect (Figure 5f). This suggests that the order of applying the two drugs is important. Several clinical trials are currently in progress to investigate the efficacy of combinatory or sequential therapies with sorafenib, although data on survival benefits are not currently available.

An important future perspective will be whether the results from this *in vitro* model can be extrapolated to the *in vivo* situation or into clinical practice for HCC, since the CSC hierarchy *in vivo* is likely more complex than that *in vitro* [36,37]. However, several studies have demonstrated that CSC markers initially identified from HCC lines *in vitro* have considerable relevance for both *in vivo* models and clinically. For example, it was shown in a xenograft model that CD13(+) cells are responsible for HCC relapse and that CD13-targeting therapy is effective [33]. Also, the prognosis for HCC patients can be stratified by the expression of AFP and the CSC marker EpCAM [47]. Expression of the CSC marker CD24 is associated with the likelihood of relapse after HCC surgery [16]. Circulating HCC cells expressing CD90(+)/CD44(+), EpCAM or ICAM have also been reported as significant predictors of HCC relapse [13,48,49]. Although CSC-targeting treatments have yet to show survival benefits for HCC patients, several preclinical trials are currently in progress to test the clinical efficacies of such therapies. We propose that our Li-7 model is worthy of further detailed investigations. In addition, this system could be used to investigate the effects of other cytotoxic drugs and to screen new drugs targeting CSCs to identify more efficient combination therapies for HCC.

## Conclusion

We identified a hepatocellular carcinoma cell line, Li-7, which has some unique features in relation to the maintenance of a clearly heterogeneous hierarchy based on the CD13(+) cancer stem cells (CSCs), and to a change in phenotype (population change) upon differentiation of the CSCs during culture. We found that sorafenib preferentially targets the CSCs *in vitro*, supporting the use of this model for screening the drugs targeting the CSCs in HCC. “Heterogeneous and unstable” cell lines might contribute more to the cancer research in the CSC era than the conventional “homogeneous and stable” cell lines.

## Additional files

Additional file 1: Figure S1. CD13 and CD166 expressions in spheroids. FACS analysis indicated that spheroid colonies produced by the bulk Li-7 cell population (left), CD13(+)/CD166(−) cells (middle) and CD13(−)/CD166(−) cells (right) are mostly composed of CD13 (+)/CD166(−) cells.
Additional file 2: Figure S2. Triple staining of CSC markers together with CD13 and CD166. Immunostaining of Li-7 cells for the CSC markers CD133(+) (left), CD44(+) (middle), and CD24(+) (right) after flow cytometry for CD13 and CD166 showed these markers are expressed in all 3 subfractions.
Additional file 3: Figure S3. Schema showing the Li-7 cell line hierarchy and the cell targets for chemotherapy.
