# Supplementary figures and images for: Identification of a unique hepatocellular carcinoma line, Li-7, with CD13(+) cancer stem cells hierarchy and population change upon its differentiation during culture and effects of sorafenib
Source: BMC Cancer. 2015 Apr 11;15:260. doi: 10.1186/s12885-015-1297-7 (PMC4396571; doi:10.1186/s12885-015-1297-7)

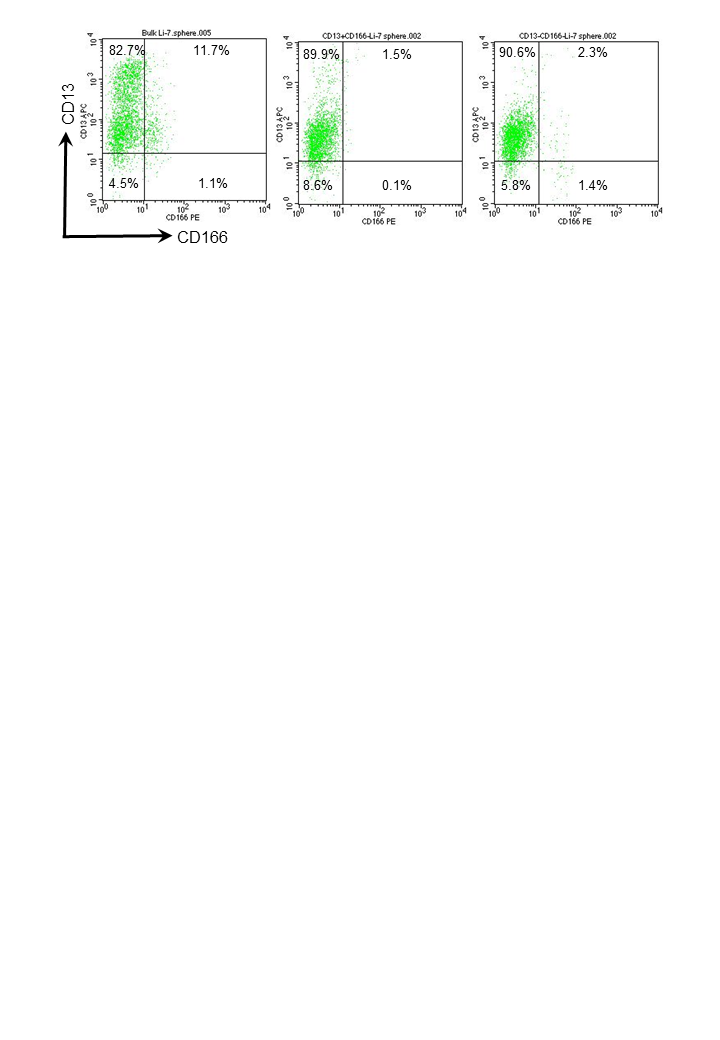

Supplement: Additional file 1: Figure S1. — CD13 and CD166 expressions in spheroids. FACS analysis indicated that spheroid colonies produced by the bulk Li-7 cell population (left), CD13(+)/CD166(−) cells (middle) and CD13(−)/CD166(−) cells (right) are mostly composed of CD13 (+)/CD166(−) cells. [file 12885_2015_1297_MOESM1_ESM.tiff]

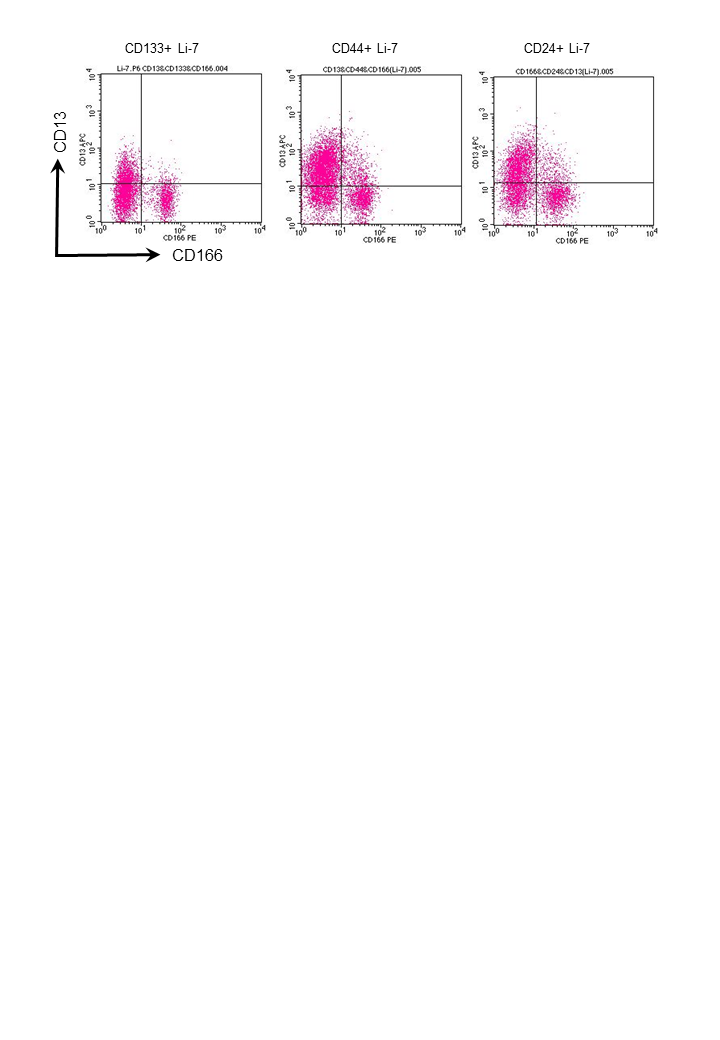

Supplement: Additional file 2: Figure S2. — Triple staining of CSC markers together with CD13 and CD166. Immunostaining of Li-7 cells for the CSC markers CD133(+) (left), CD44(+) (middle), and CD24(+) (right) after flow cytometry for CD13 and CD166 showed these markers are expressed in all 3 subfractions. [file 12885_2015_1297_MOESM2_ESM.tiff]

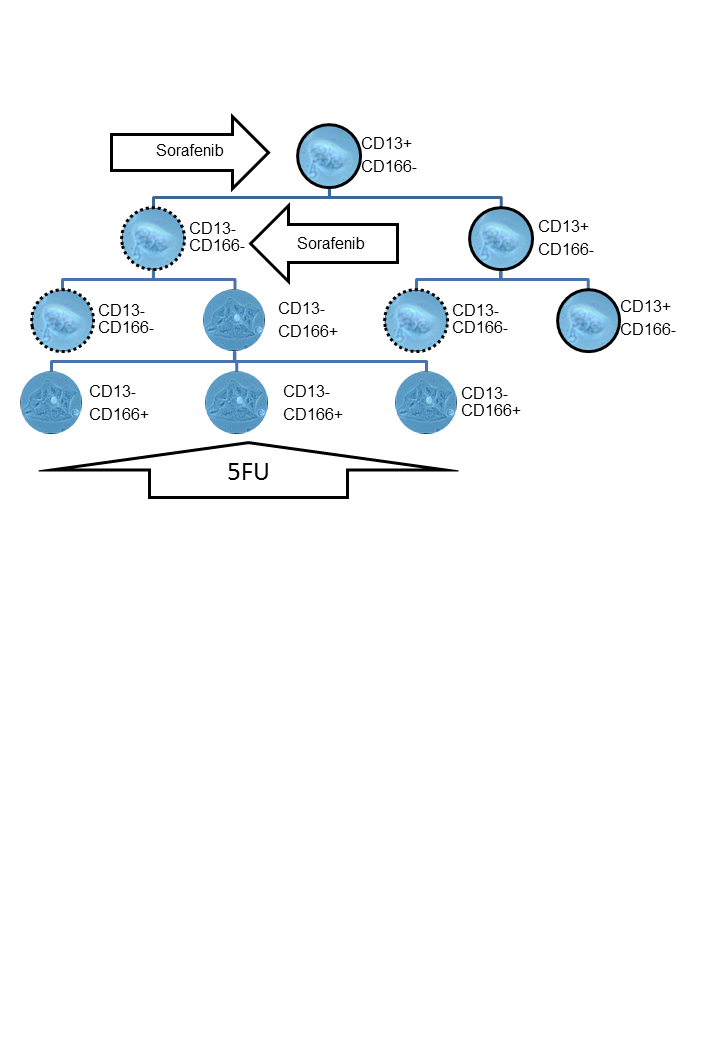

Supplement: Additional file 3: Figure S3. — Schema showing the Li-7 cell line hierarchy and the cell targets for chemotherapy. [file 12885_2015_1297_MOESM3_ESM.tiff]
